# A new record of *Potamanthellus caenoides* Ulmer 1939 (Ephemeroptera: Neoephemereidae) from the southern Western Ghats of India

**DOI:** 10.3897/BDJ.3.e5021

**Published:** 2015-05-08

**Authors:** C. Selvakumar, K.G. Sivaramakrishnan, S. Janarthanan

**Affiliations:** ‡ Department of Zoology, University of Madras, Guindy Campus, Chennai-600025, India; § Flat 3, Door No. 7, Gokulam Apartments, Gokulam Colony, West Mambalam, Chennai-600033, India

**Keywords:** Ephemeroptera, new record, *Potamanthellus caenoides*, southern Western Ghats, India

## Abstract

**Background:**

As part of ongoing exploration of the mayflies of hill streams of the southern Western Ghats of India, we establish a new record of mayfly.

**New information:**

*Potamanthellus caenoides* Ulmer 1939 is newly recorded based on larval collection from the upstream of Silent Valley National Park of the southern Western Ghats. Brief ecological notes are appended.

## Introduction

Ephemeroptera is a biogeographically significant archaic order of aquatic insects abounding in several enigmatic families in the pantropical region, especially in the Oriental Realm. Neoephemeridae is a small group of mayflies presently confined to Holartic and Oriental regions. Bae 1998 recognized only three genera viz., *Potamanthellus* Lestage 1930 (seven species), *Neoephemera* McDunnough 1925 (five species) and *Ochernova* Bae 1998 (one species), synonymising *Leucorhoenanthus* Lestage 1930 (one species) with *Neoephemera*. This is not accepted by Kluge 2004 and Bauernfeind and Soldán 2012. Larvae of Neoephemeridae have unique operculate gills on the second abdominal segment that are fused medially. The larvae of *Potamanthellus* are distinguished from those of *Neoephemera* and *Ochernova* by their densely setate mouthparts, by their lack of well developed lateral expansions of the pronotum and mesonotum, and by their possession of rows of long setae on the caudal filaments (Bae 1998). However, the larvae of *Potamanthellus* cannot be differentiated from *Leucorhoenanthus* by any plesiomorphic or appmorphic larval traits. Based on our larval collections of Ephemeroptera of the Western Ghats, we establish a new record of *Potamanthellus caenoides* (Ulmer 1939), which is a significant range extension to south Asia from its known range viz., Sumatra Island located in Southeast Asia. Differential diagnosis of *P. caenoides* is verified based on the larval descriptions given (Ulmer 1939) and subsequent revisionary studies (Bae 1998). Brief ecological notes are appended.

## Materials and methods

The material used for this study was collected from the up-streams of Silent Valley National Park in south western region of the Western Ghats of peninsular India. The specimens were preserved in 85% ethanol. Some specimens were mounted on slides to enable detailed microscopic observations. Photographs were taken on the stereozoom and brightfield microscopes (Magnus and Nikon Eclipse 80i).

## Taxon treatments

### Potamanthellus caenoides

Ulmer 1939

#### Materials

- **Type status:** Other material. **Occurrence:** individualCount: 7; sex: male & female; lifeStage: Larva; **Taxon:** kingdom: Animalia; phylum: Arthropoda; class: Insecta; order: Ephemeroptera; family: Neoephemeridae; genus: Potamanthellus; specificEpithet: caenoides; taxonRank: species; taxonomicStatus: accepted; **Location:** country: INDIA; stateProvince: Kerala; municipality: Silent Valley National Park; locality: Poochipara; verbatimElevation: 935 m; verbatimLatitude: 11°06’49.5” N; verbatimLongitude: 76°25’52.4” E; **Identification:** identifiedBy: C. Selvakumar & K. G. Sivaramakrishnan; **Event:** samplingProtocol: Hand picking; year: 2013; month: April; day: 18; habitat: Cascade

#### Diagnosis

**Figure 1.**
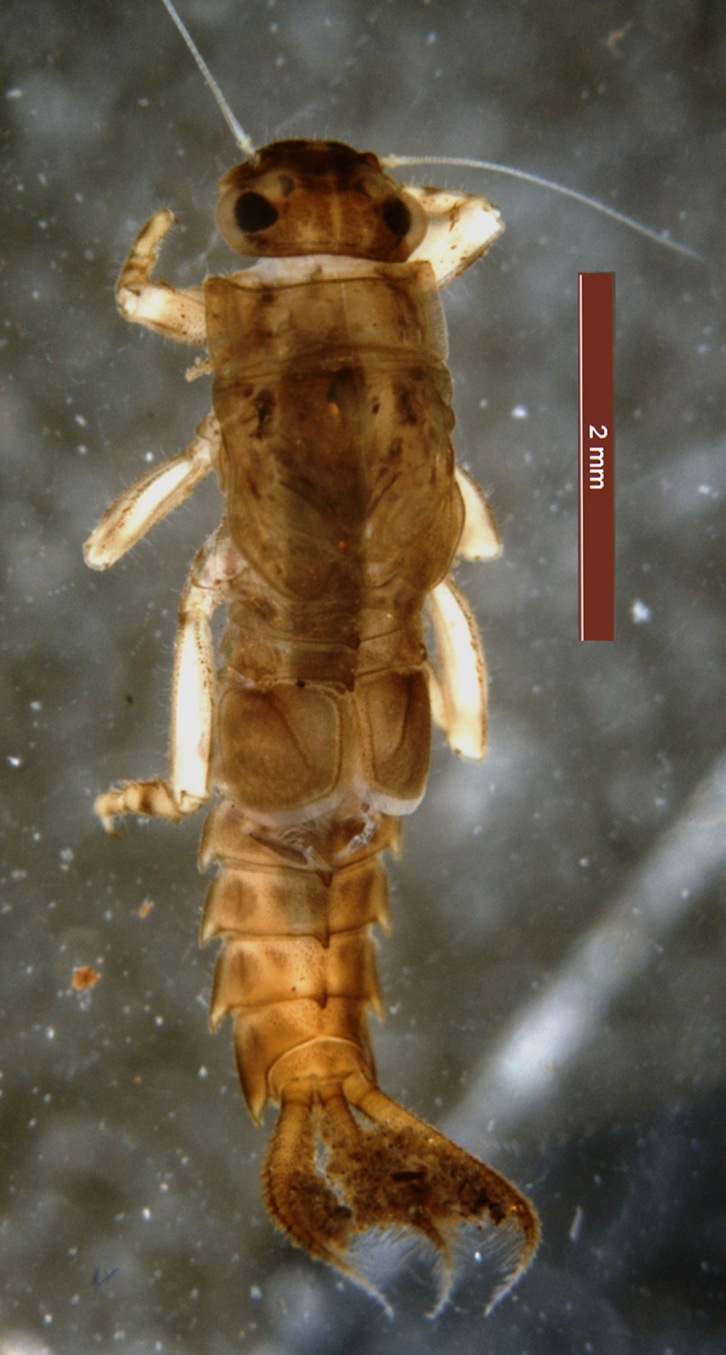
Dorsal view of *Potamanthellus caenoides* Ulmer 1939.

*Potamanthellus caenoides* is distinguished from other species of *Potamanthellus* by the following combination of characters in larvae: (i) a distinct diagonal ridge on operculate gills (Fig. 1); (ii) distinct tubercles on abdominal terga 6–8 (Fig. 1); (iii) dorsal forefemora with transverse row of setae (Fig. 2); (iv) relatively small body size (<8 mm) (Fig. 1) and (v) relatively short caudal filaments that possess strongly developed lateral setae (Fig. 1). *P. caenoides* is distinguished from closely related species *P. ganges* by the following characters: (i) posteromedian tubercle on abdominal terga 1–2 and 6–8 distinct (Fig. 1); (ii) rows of hairlike setae strongly developed and mature body ca. 6-8 mm (Fig. 1) and (iii) dorsal forefemora with transverse row of setae (Fig. 2).

#### Distribution

Indonesia (Sumatra (Ulmer 1939), Java, Bali, Lombok and Flores), Malaysia (Malay peninsula, Sabah and Sarawak), Philippines (Mindanao), Thailand (Bae 1998), Vietnam (Nguyen and Bae 2003) and India (southern Western Ghats).

#### Biology

The larvae of *P. caenoides* occur in moderately fast flowing mountain streams and rivers ranging 850-935 m in altitude. The streams and rivers are canopied by predominant riparian trees. The substrates consist of relatively coarse particles (boulder 30%, cobble 20%, pebble 20% and gravel and sand 30%), fallen leaves and detritus. The water temperature in April ranges 18-23°C. Larvae were collected by Kick samples and hand picking.

#### Taxon discussion

Presently this genus consists of seven species viz. *P. amabilis* (Eaton 1892), *P. caenoides* (Ulmer 1939), *P. chinensis* (Hsu 1936), *P. edmundsi* (Bae 1998), *P. ganges* (Bae 1998), *P. shaowuensis* (Gui et al. 1999) and *P. unicutibius* (Nguyen and Bae 2003). However, only one species viz., *P. ganges* is known from India from the tributary of Ganges (Bae 1998). *P. caenoides* (Ulmer 1939) is new record from the southern Western Ghats and second species from India.

## Supplementary Material

XML Treatment for Potamanthellus caenoides

## Figures and Tables

**Figure 2a.**
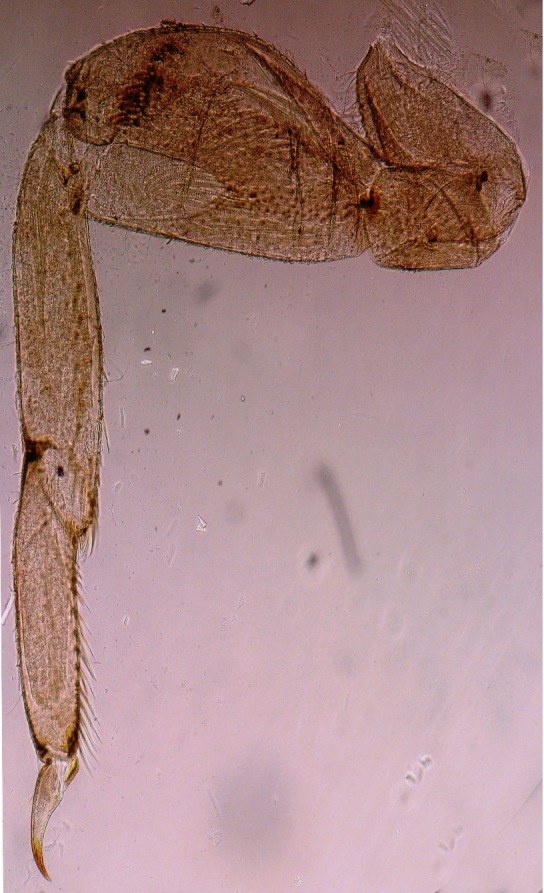
Foreleg

**Figure 2b.**
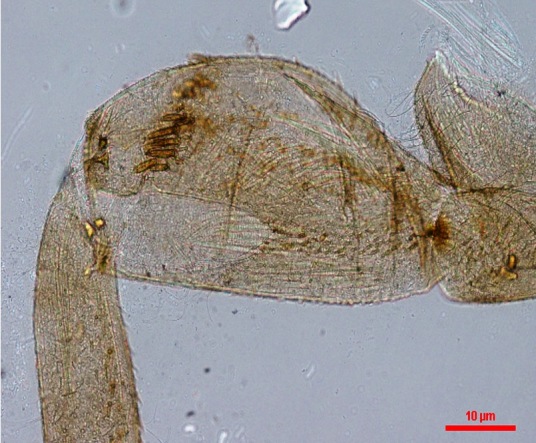
Dorsal forefemora with transverse row of setae
